# Anticancer compounds from *Streptomyces*: insights from metagenomics and mechanistic perspective

**DOI:** 10.1007/s12223-025-01332-x

**Published:** 2025-10-03

**Authors:** Muhanna Mohammed Al-shaibani, Noraziah Mohamad Zin, Juwairiah Remali, Nik Marzuki Sidik, Nabil Ali Al-Mekhlafi, Vanitha Mariappan, Asif Sukri

**Affiliations:** 1 Center for Diagnostic, Therapeutics and Investigative Studies, Faculty of Health Sciences, Universiti Kebangsaan Malaysia, 50300 Kuala Lumpur, Malaysia; 2 Institute of Systems Biology (INBIOSIS), Universiti Kebangsaan Malaysia, 43600 Bangi, Selangor Malaysia; 3 Faculty of Agro-Based Industry, Universiti Malaysia Kelantan, 17600 Jeli, Malaysia; 4 Biochemical Technology Program, Department of Chemistry Faculty of Applied Science, Thamar University, P.O. Box 87246, Thamar, Yemen; 5 Center for Toxicology and Health Risk Studies (CORE), Faculty of Health Sciences, Universiti Kebangsaan Malaysia, Jalan Raja Muda Abdul Aziz, 50300 Kuala Lumpur, Malaysia; 6 Department of Biological Sciences and Biotechnology, Faculty of Science and Technology, Universiti Kebangsaan Malaysia, 43600 Bandar Baru Bangi, Malaysia

**Keywords:** Metagenomics, *Streptomyces*, Anticancer compound

## Abstract

Cancer continues to be a leading cause of death globally, driving the ongoing search for novel bioactive compounds with therapeutic potential. Metagenomic sequencing has revolutionized this pursuit by enabling the direct detection and genomic assembly of previously uncultured *Streptomyces* species from environmental DNA, circumventing traditional cultivation limitations. This review explores recent advances in metagenomics-driven discovery of anticancer compounds derived from *Streptomyces*, with a focus on identifying biosynthetic gene clusters (BGCs) responsible for producing bioactive secondary metabolites. Over the past decade, metagenomic approaches have been adopted to uncover new species of *Streptomyces* and anticancer compounds. Although metagenomics has been adopted in research and discovery of new *Streptomyces*, its application in the discovery of *Streptomyces*-related pathways pertaining to anticancer compounds remains limited. Furthermore, clinical translation remains limited, highlighting the need for further research. By examining metagenomic methodologies and the mechanisms of action of these compounds, this review provides an updated and focused perspective on *Streptomyces*-derived anticancer agents and their potential for future drug development.

## Introduction

*Streptomyces* is a genus of Actinobacteria, a group of gram-positive bacteria that form branching filaments. They are known for their ability to produce a wide variety of secondary metabolites, including antibiotics, anticancer compounds, and enzymes (Al-Shaibani et al. 2021; Baba et al. 2021a, b). *Streptomyces* spp. are commonly found in soil and decomposed plant material, where they act as decomposers. They are also known to form symbiotic relationships with plants, including the production of anti-microbial compounds to protect the plants against other microorganisms (Chandra and Kumar 2017; Mazlan et al. 2020; Baba et al. 2021a, b). The typical morphology of *Streptomyces* is that of long, filamentous, branching cells that form aerial hyphae and substrate mycelia. They also produce spores on specialized structures called sporangia. The discovery of *Streptomyces* was a significant event in the field of antibiotics, as many of the first antibiotics discovered were made by this microorganism, including streptomycin, tetracyclines, and many others (Quinn et al. 2020; Ahmad et al. 2017; Zin et al. 2017). It is possible to discover more bioactive compounds from *Streptomyces* spp. as anticancer compounds.

The genome of *Streptomyces* is typically large and complex, with a high number of genes and a high degree of genetic diversity. The genome size varies among different *Streptomyces* spp., with some having genomes as large as 16 million base pairs and others having genomes as small as 4 million base pairs (Dinesh et al. 2017). It is known that the genome contains high GC content (typically around 70%), a high proportion of non-coding DNA, such as introns and intergenic regions, as well as a high number of repetitive elements (Peterson and Kaur 2018). Secondary metabolic gene clusters are responsible for the biosynthesis of a wide range of biologically active compounds in *Streptomyces* spp. (Remali et al. 2017). These compounds are not essential for the growth and survival of the organism but are important for its ecological niche and interactions with other organisms with a wide range of biological activities, such as antibacterial, anticancer, anti-inflammatory, and anti-viral activities (Qi et al. 2019). The biosynthesis of these compounds is typically regulated by complex genetic and environmental factors, and the genes responsible for their biosynthesis are often organized into clusters that are co-regulated and co-transcribed (Baba et al. 2021a, b). These secondary metabolic gene clusters are often large and contain dozens or even hundreds of genes often found in specific regions of the genome (Remali et al. 2017; Droste et al. 2020). Hence, *Streptomyces* spp. serves as prolific producers of diverse secondary metabolites, making them valuable hosts for library construction. However, in complex metagenomes, the presence of endogenous genes in *Streptomyces* spp. may interfere with the expression of heterologous gene clusters, and due to a vast biosynthetic capacity, they might not express all gene clusters from environmental sources efficiently. Recent reviews have explored the role of *Streptomyces* in the discovery of antibiotic and anticancer compounds (Alam et al. 2022; Lee et al. 2020; Sethi et al. 2024). However, these studies have not specifically focused on the application of metagenomics in the identification of anticancer agents. In contrast, this review centers on metagenomic strategies for uncovering novel *Streptomyces-*derived compounds with anticancer activity, setting it apart from previous literature (e.g., Sethi et al. 2024). Unlike earlier work that primarily emphasizes antibiotic biosynthesis (Sivalingam et al. 2024), our review emphasizes genome mining, bioinformatics tools, and the functional analysis of biosynthetic gene clusters revealed through metagenomics. We aim to provide an updated and focused perspective on how metagenomic technologies have revolutionized the exploration of *Streptomyces* species and their biosynthetic potential for anticancer drug discovery.

### Metagenomics and *Streptomyces*-derived anticancer compounds

The metagenomics approach allows the identification and characterization of biosynthetic gene clusters from uncultivated microorganisms, which can potentially produce novel natural products. This current approach involves studying genetic material directly obtained from clinical or environmental samples using DNA sequencing. It is used to study the diversity and abundance of microorganisms in different environments, such as soil, water, and air (Liu et al. 2025). This can help researchers understand the role of microorganisms in ecosystem functioning and identify potential bioremediation agents (Nowrotek et al. 2019). In new anticancer compound discovery, recent study identified the biosynthetic pathway of lasonolide A from marine sponge using metagenomic analysis of sponge microbiome (Uppal et al. 2022).

Metagenomics is able to uncover microbial diversity that is difficult to culture in the laboratory. In addition to its ecological applications, metagenomics has many other usages, including in the fields of energy, food and nutrition, agriculture, biosensors, medicine, biotechnology, and industrial applications (Lema et al. 2025). For instance, metagenomics can help identify new enzymes and bioactive compounds with potential applications in various fields (Priscilla et al. 2025). Furthermore, by studying the genomes of microorganisms in different contexts, metagenomics can help in identifying the functional roles of genes and pathways and ultimately contribute to a better understanding of biology and biotechnology. It can also be used to discover new bioactive compounds from *Streptomyces* spp. by identifying and characterizing the genes responsible for the biosynthesis of these compounds (Mahapatra et al. 2020; Iqbal et al. 2016; Nor Azlan et al. 2021).

### Metagenomics exploration in the research and discovery of* Streptomyces*

Metagenomic has been used to identify the essential *Streptomyces* from various environmental sources. Recent study uncovered richness of Actinobacteria from Gobi Desert with potential antibiotic producing metabolites, of which most of them belong to *Streptomyces* species (Yang et al. 2024). Although they noted the presence of antibiotic producing pathways in *Streptomyces*, the *Streptomyces* detected are unculturable and complicate the potential bioprospecting purpose. Besides that, metagenomic analysis of hospital wastewater reveals the abundance of *Streptomyces* with potential production of antimicrobial compounds (Lema et al. 2025). Studies that directly examined the production of anticancer compounds in *Streptomyces* from metagenomic sequences are limited. It is challenging to identify anticancer compounds directly from metagenomic sequences because metagenomic sequences usually map to diversity of microbes. While metagenomic can uncover the presence of biosynthetic gene cluster of anticancer compounds, the application is limited as it does not reveal whether the compounds are produced or not. However, Khan et al. (2023) identified *Streptomyces* abundance in pancreatic tumor tissue with positive prognosis compared to that of negative prognosis, suggesting the potential production of unidentified anticancer compounds of *Streptomyces*.

Compounds produced by *Streptomyces* have been used as chemotherapeutic agents for the treatment of a variety of cancers, with actinomycins being the most commonly used (Alam et al. 2022). Studies that examine the production of compounds or identification of *Streptomyces* from environment with potential as anticancer agent are limited. Heinrich et al. (2020) adopted metagenomic sequencing to profile sediment collected from Stellwagen Bank National Marine Sanctuary and discovered biosynthetic gene cluster that mapped to *Streptomyces scabrisporus* secondary metabolite production. Recent study uncovered *Streptomyces* clone, namely PS49, isolated from metagenomic library that produces strong anticancer compound (8-demethoxy-10-deoxysteffimycin) against skin cancer (Sujith et al. 2024). Microbiome and metagenomic profiling of ginseng soils uncovered diverse *Streptomyces* genus with biosynthetic gene clusters that play an essential role to encode antimicrobial compounds and essential natural products with health benefits. Metagenomic study later guided the researchers to isolate *Streptomyces*, namely isolates WY33, WY141, and WY144, that possessed strong antifungal activity (Huang et al. 2024). In summary, our literature search reveals lack of studies conducted on discovery of anticancer compounds from *Streptomyces* using metagenomic analysis.

Although metagenomic application in discovery of anticancer compound from *Streptomyces* is limited, studies have reported the successful isolation of over 30 compounds from different *Streptomyces* spp. strains as shown in Tables 1 and 2 (Law et al. 2020).

**Table 1:** List of important *Streptomyces* sp. and its activity isolated between 2015 and 2022

Name of *Streptomyces*	Location of collections	Activity and compound name	References
*S. lividans*	Soil and decaying plant material	Lividomycin Antibacterial and anticancer	(Rebets et al. 2017; Ogawara 2018)
*S. verticillus*	Various environments across the world	Verticillin, Bryostatin-1 and ET-743	(Mohan et al. 2022; Palmano et al. 2000)
*S. spp. ADR1*	Soil collected from the Algerian Sahara Desert	Β-And-T Rubromycin Anticancer, antibiotic	(Boumehira et al. 2022)
*S. spp. OA293*	Coast of China, The Mediterranean, and the Atlantic Ocean	Urdamycin E and Urdamycin V Anticancer	(Dan et al. 2020)
*S. spp. KCTC 0041BP*	Soil sample in Cheolwon, Republic of Korea	Linearmycins Anticancer, antibiotic	(Nguyen et al. 2021)
*S*. SH4 and SH12	Soil samples Collected from Beni-Suef Governorate, Egypt	Exhibited potent anticancer activity against the hepatoma cell line hepatoma G2 (Hepg2)	(Osama et al. 2022)
*S. parvulus*	Soil samples collected from a desert ecosystem in Oman	Potential anticancer activity	(Xue et al. 2019)
*S. roseosporus*	Soil samples collected from a Mediterranean ecosystem	Potential anticancer activity	(Ruthledge and Challis 2015)
*Streptomyces* clone PS49	Mangrove sediment	Isolation of 8-demethoxy-10-deoxysteffimycin with potential anticancer activity against skin cancer	(Sujith et al. 2024)

**Table 2:**
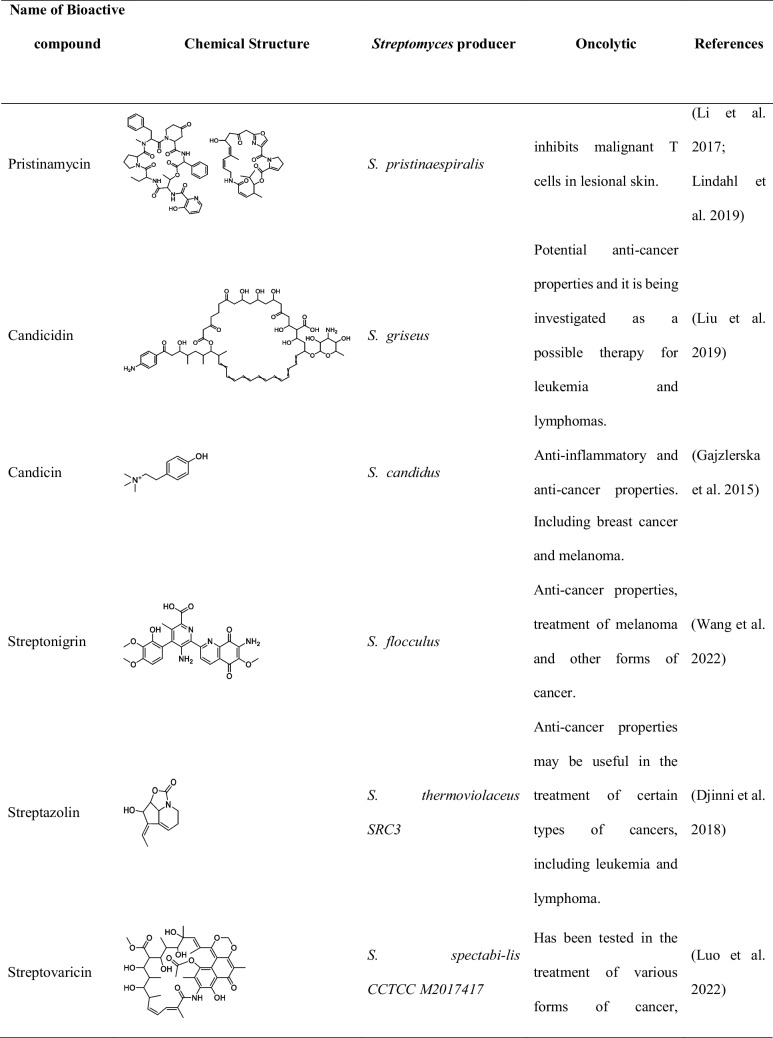

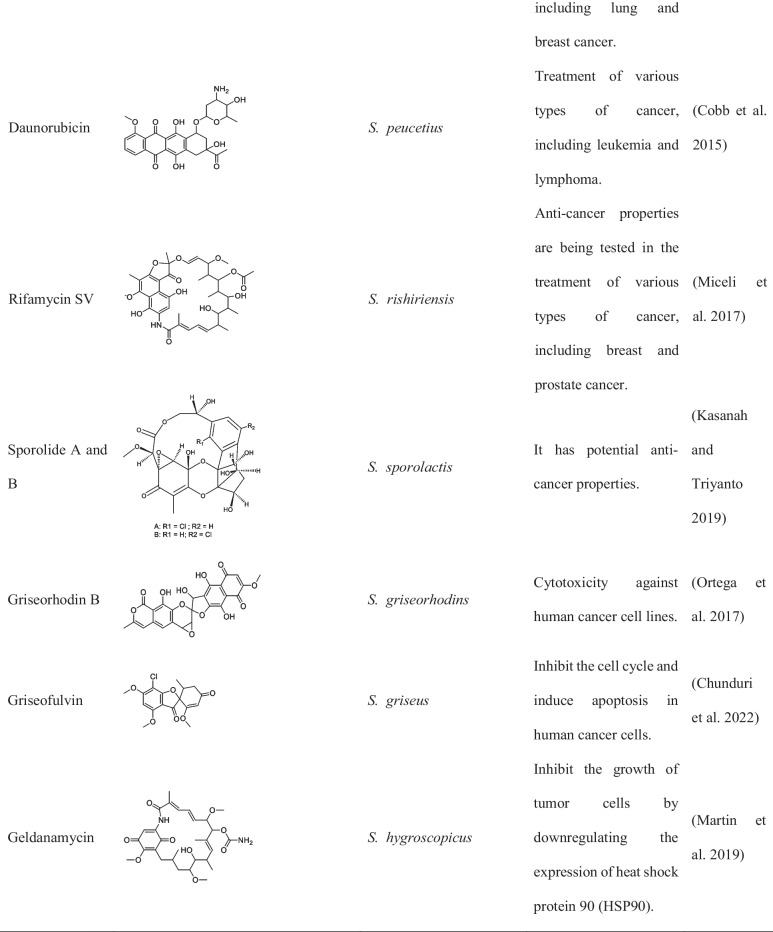

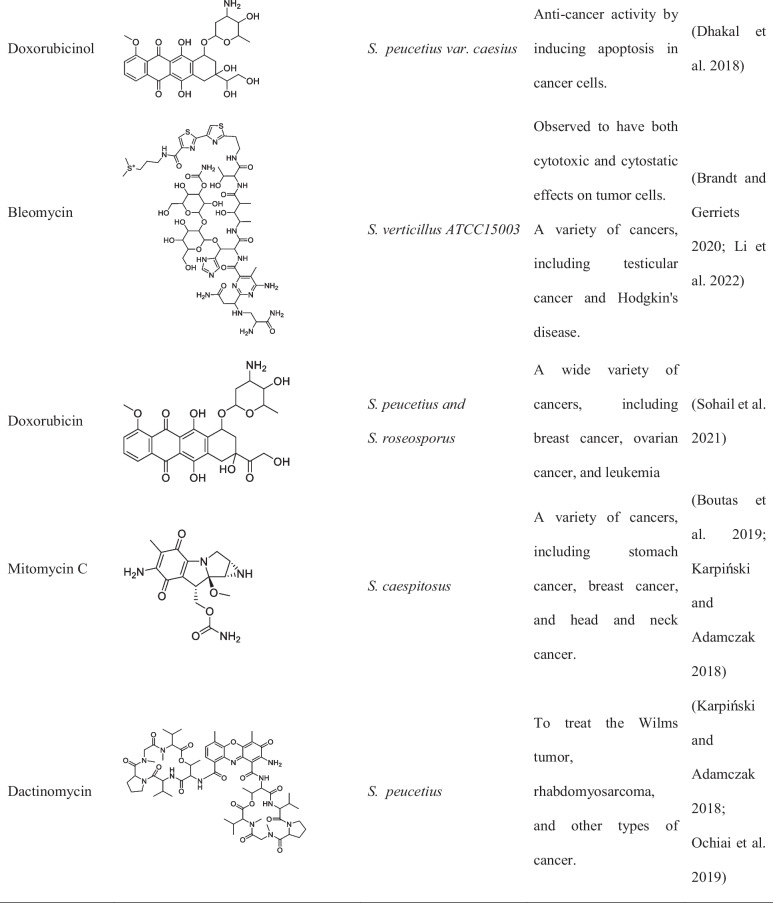

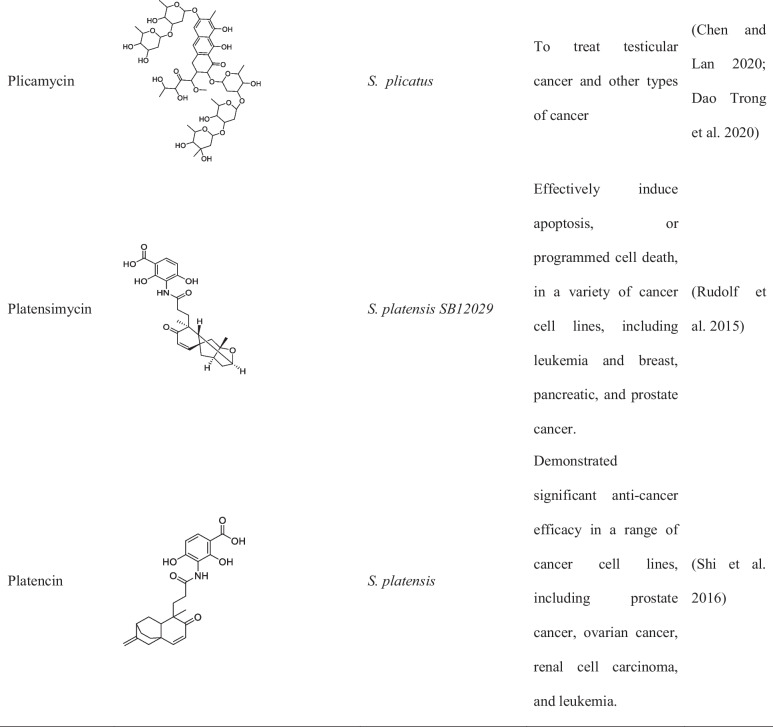
Names, chemical structure, and uses of anticancer compounds isolated from *Streptomyces* spp

### Mechanism of action of some anticancer bioactive compounds isolated from *Streptomyces*

**Fig. 1:**
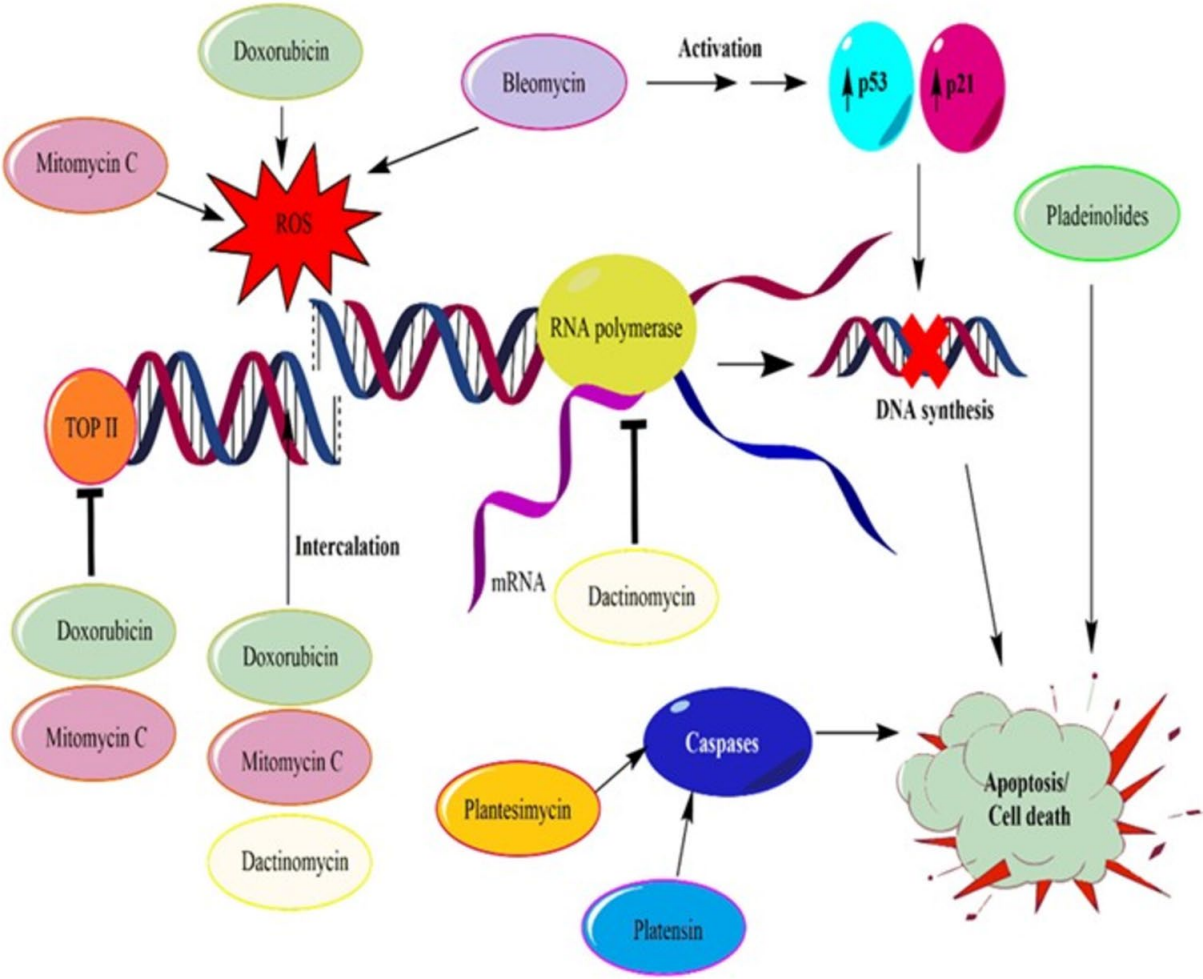
Mechanism of action of anticancer compound isolated from *Streptomyces* spp. The mechanism involved inhibition of DNA synthesis such as inhibition of topoisomerase II (TOP II) and DNA intercalation which prevent normal replication and transcription, formation of reactive oxygen species (ROS), activation of tumor suppressor protein such as p53 and p21, inhibition at RNA polymerase as well as activation of caspases which induced apoptosis. This figure is produced by the authors

Our literature search uncovered the mechanism of action of several cancer bioactive compounds isolated from *Streptomyces*, including bleomycin (BLM), doxorubicin (DXR), mitomycin C, pladienolides (Plad), platensimycin (PTM), and platencin (PTN). While these compounds have shown anticancer activity, their full therapeutic potential has not been completely studied or tested in human clinical trials. The mechanism of action of anticancer bioactive compounds isolated from *Streptomyces* has been well-documented for several decades. Many of these compounds, such as DXR and mitomycin C, are widely used in clinical settings. These agents act primarily through mechanisms such as DNA intercalation, topoisomerase inhibition, and apoptosis induction in cancer cells. While ongoing research explores new derivatives and improved formulations, their established role in oncology remains critical. As such, their clinical applications remain uncertain (Shi et al. 2016; Fedorenko et al. 2015; Liu 2016). Nonetheless, the review provides a detailed discussion of the mechanisms of action of these compounds, which can provide insights into their potential therapeutic applications in the future. By understanding the molecular targets and pathways of these compounds, researchers can develop more effective treatments for cancer and other diseases (Fig. 1).

### Bleomycin

BLM, originally discovered by Umezawa et al. (1966) from *Streptomyces verticillus*, has been widely used as an anticancer agent (Bolzán and Bianchi 2018). Despite its efficacy, its clinical application is limited by severe side effects, including pulmonary fibrosis and anaphylaxis. BLM, isolated from *S.* *verticillus* *ATCC15003* (Umezawa et al. 1966; Li et al. 2022), is a chemotherapy drug that works by inhibiting DNA synthesis in cancer cells (Bolzán and Bianchi 2018). BLM primarily consists of bleomycin A_2_ and B_2_. Analysis of BLM chemical structure shows that it consists of a pyrimidine ring that links to thiazole and methylvaleric acid moiety important in binding metal ions for DNA cleavage. In addition, BLM is amphipathic which facilitates its interaction with DNA and cell membrane (Bolzán and Bianchi 2018). The exact mechanism of action is not fully understood, but it is thought to bind and break the DNA strands, causing the cancer cells to die. It also generates free radicals that cause damage to the cell membrane and proteins (Della Latta et al. 2015). BLM forms complexes with metal ions, like Fe(II) which undergo oxidation to form BLM-Fe(III), which can lead to the generation of hydroxyl radicals. These hydroxyl radicals can cause damage to the DNA by breaking the phosphodiester bonds between the nucleotides (Murray et al. 2018). This results in the formation of double-stranded breaks in the DNA which are difficult to repair, leading to cell death. BLM can activate p53 and p21, two tumor suppressor proteins that are involved in cell cycle regulation, which also contribute to the death of cancer cells (Su et al. 2017). It is important to note that BLM also has some side effects, such as skin and lung toxicity, which need to be monitored during treatment (Watson et al. 2018).

Due to these risks, its use requires careful monitoring, and cumulative dosing is often limited to reduce lung toxicity (Della Latta et al. 2015).

Recent research has explored novel drug delivery systems, such as nanoparticle-based formulations, to enhance BLM’s therapeutic index while reducing systemic toxicity (Salehan et al. 2025). Additionally, combination therapy with protective agents such as amifostine has been investigated to mitigate lung toxicity while preserving anticancer efficacy (Yang et al. 2023).

### Doxorubicin

DXR isolated from *S. peucetius var. caesius* is a chemotherapy drug that interferes with the DNA of cancer cells, leading to their death (Rawat et al. 2021). Primary chemical structures of DXR include an anthracycline core that is essential in DNA intercalation and damage, danosamine sugar for cytotoxic activity, and carbonyl and hydroxyl groups that enhance the interaction of this anticancer compound with DNA (Rawat et al. 2021). The exact mechanism of action of DXR is not fully understood, but it is believed to react by inhibiting the activity of an enzyme called topoisomerase II. Topoisomerase II is an enzyme that helps the DNA unwind during replication and transcription. DXR binds to the topoisomerase II enzyme, preventing it from functioning properly (Varela-López et al. 2019). This leads to the accumulation of DNA damage in the cancer cells, resulting in cell death. DXR also intercalates into the DNA, which causes the formation of adducts, leading to DNA damage and cell death (Bhagat and Kleinerman 2020). DXR can also cause the accumulation of reactive oxygen species (ROS) which can lead to apoptosis and cell death (Christidi and Brunham 2021). It is important to note that DXR can also have side effects, such as cardiotoxicity and myelosuppression, which need to be monitored during treatment (Basak et al. 2021). DXR is a well-established chemotherapeutic agent used in the treatment of various cancers, including breast cancer, lymphoma, and leukemia. However, its clinical application is significantly limited by severe cardiotoxicity, which can lead to irreversible heart failure (Rawat et al. 2021). The mechanism of DXR-induced cardiotoxicity involves the generation of reactive oxygen species (ROS) and mitochondrial dysfunction, leading to apoptosis in cardiac cells. Additionally, DXR is associated with myelosuppression, nausea, vomiting, and increased risk of secondary malignancies (Kamińska et al. 2023). Strategies to mitigate these adverse effects, such as liposomal formulations and cardioprotective agents like dexrazoxane, have been explored to enhance their therapeutic index (Basak et al. 2021).

### Mitomycin C

Mitomycin C is another anticancer compound that has been isolated from the *Streptomyces caespitosus.* Its chemical structure includes aquinone ring that undergoes reduction under hypoxic conditions and an aziridine ring for alkylation reaction and carbamoyl group (Botrus et al. 2022). It is a chemotherapy drug that inhibits DNA synthesis in cancer cells, leading to their death. The exact mechanism of action of mitomycin C is also not fully understood, but it is believed to crosslink the DNA strands, preventing replication and transcription (Botrus et al. 2022). Mitomycin C is an alkylating agent that forms covalent bonds with the DNA bases, particularly guanine and adenine. This crosslinking of the DNA strands leads to the formation of DNA-DNA or DNA–protein crosslinks, which prevents the normal replication and transcription of the DNA (Zacarias et al. 2021). Mitomycin C can also cause the formation of ROS which can lead to DNA damage and apoptosis (Chaudhry et al. 2021). Mitomycin C can also inhibit the activity of topoisomerase II, an enzyme that helps the DNA to unwind during replication and transcription, like DXR which leads to the accumulation of DNA damage in the cancer cells, resulting in cell death (Sarker et al. 2020). Mitomycin C is a well-established chemotherapeutic agent derived from *S. caespitosus*. Unlike DXR, it functions as a DNA crosslinking agent, inhibiting DNA replication in rapidly dividing cancer cells. It has been widely used in treating gastrointestinal and bladder cancers. However, its application is limited by severe myelosuppression and potential nephrotoxicity. Recent advancements focus on optimizing its delivery to reduce systemic toxicity (Rodriguez-Reyes et al. 2019).

### Dactinomycin

Dactinomycin, also known as actinomycin D, isolated from *S. peucetius* (Karpiński and Adamczak 2018), is a chemotherapy drug that inhibits DNA synthesis in cancer cells, leading to cell death (Shaik et al. 2022). Chemical structure of dactinomycin shows that it consists of a phenoxazinone chromophore that plays an essential role in DNA intercalation and two cyclic pentapeptide lactone rings that stabilizes DNA binding. The precise mechanism of dactinomycin is not fully understood, but it is thought to intercalate into the DNA between the base pairs of DNAs, particularly adenine (A) and thymine (T), inhibiting the activity of RNA polymerase (Sarker et al. 2020). This intercalation causes the DNA to twist and bend, which prevents the process of replication and transcription of the DNA. It also causes the formation of DNA-dactinomycin adducts which prevents the normal replication and transcription of the DNA. On the other hand, dactinomycin specifically inhibits RNA polymerase activity in the early stages of transcription, thus inhibiting the formation of the RNA chain, and protein synthesis (Prylutskyy et al. 2015; Das et al. 2017). It is important to note that dactinomycin can also have side effects, such as myelosuppression, which need to be monitored during treatment (Sharami and Saffarieh 2020).

### Pladienolides

Plad are a class of compounds produced by some *S. platensis* that are well known as anticancer compounds (Murphy et al. 2022). These compounds belong to the class of polyketides and have a complex polycyclic structure. Its chemical structure consists of a 12-membered macrolide ring, conjugated diene system, and side chains and functional groups. Plad have been recognized as a potent anticancer molecule for nearly two decades. These compounds exert their effects by inhibiting the spliceosome machinery, thereby disrupting RNA splicing in cancer cells. Their clinical potential has been extensively studied, and ongoing research focuses on optimizing their therapeutic applications and addressing bioavailability challenges (Eymin 2021). Plad have been shown to inhibit the growth of a wide range of cancer cell lines in laboratory studies and have been shown to have activity against several types of cancer, such as melanoma, ovarian cancer, and lung cancer (Zhang et al. 2019). This anticancer compound has been found to have multiple mechanisms of action, including the inhibition of cell proliferation, the induction of apoptosis, and the modulation of signaling pathways involved in cancer (Jorge et al. 2020). They have also been shown to have anti-angiogenic properties and to inhibit the growth of tumor blood vessels, which is an important aspect of cancer progression (Zhang et al. 2019). Phase I clinical trial of pladienolide B derivative, namely E7107, in patients with advanced tumor showed that this drug exhibited adverse effects to some patients that include reversible vision loss and gastrointestinal symptoms (Eskens et al. 2013). Despite promising results of Plads in preclinical studies, more studies in clinical trials should be conducted to unravel the effect of this drug to humans.

### Platensimycin

PTM is a class of compounds that have been discovered from *S. platensis* SB12029 and have been studied for their potential anticancer properties (Rudolf et al. 2015). These compounds are natural product antibiotics and have a complex polyketide structure (Shi et al. 2016). PTM has been found to inhibit the growth of a wide range of cancer cell lines in laboratory studies and has been shown to have activity against several types of cancer including leukemia, melanoma, and non-small cell lung cancer (Singh et al. 2020). PTM has been found to have multiple mechanisms of action, such as inhibiting the activity of enzymes that are necessary for the survival of cancer cells and inducing cell death by activating caspases (Patra and Gasser 2017). They have also been shown to modulate the activity of signaling pathways that are important for cancer cell survival and proliferation (Menendez and Lupu 2017). Like Plads, PTM has not yet been extensively studied in vivo. However, the results of laboratory studies have provided a strong rationale for further development of PTM as a potential anticancer therapy. The discovery of PTM and their potential anticancer properties highlights the importance of investigating natural products as a source of new cancer treatments and the potential of *Streptomyces* as a rich source of biologically active compounds (Kalkreuter et al. 2020).

### Platencin

PTN is a compound that was discovered from a strain of *S. platensis* (Parsons et al. 2015). It has been studied for its potential anticancer properties. PTN has been found to inhibit the growth of a wide range of cancer cell lines in laboratory studies, including melanoma, ovarian cancer, and lung cancer. PTN has been found to have multiple mechanisms of action, such as inducing cell death by activating caspases and inhibiting the activity of enzymes that are necessary for the survival of cancer cells (Teijaro et al. 2019). It has been found to modulate the activity of signaling pathways that are important for cancer cell survival and proliferation and to inhibit angiogenesis, which is the formation of new blood vessels that supply nutrients to tumors. Like PTM, PTN has not yet been extensively studied in in vivo, especially in animal models or in human clinical trials, and more research is needed to evaluate its safety and efficacy in treating cancer. However, the results of laboratory studies have provided a strong rationale for further development of PTN as a potential anticancer therapy.

### Current challenges and future directions

Future research on *Streptomyces*-derived anticancer compounds should focus on structural modifications, targeted drug delivery, and combination therapies to enhance efficacy and reduce toxicity. Structural modifications involve optimizing the chemical composition of these compounds to improve their stability, selectivity, and potency while reducing side effects. Despite the vast potential of *Streptomyces*-derived anticancer compounds, several challenges must be addressed. Many of these compounds, while effective, have severe adverse effects that limit their clinical applications. Improved formulations, such as nanoparticle-based delivery, may enhance bioavailability and reduce toxicity.

Advances in medicinal chemistry and synthetic biology can help refine molecular structures to enhance their interaction with cancer targets while minimizing toxicity to healthy cells. Resistance mechanisms in cancer cells can also reduce the long-term efficacy of these compounds. Future approaches include nanoparticle-based delivery, antibody–drug conjugates, and combination with DNA repair inhibitors like PARP inhibitors. Overall, integrating synthetic biology, AI-driven drug discovery, and precision medicine will be crucial in refining these natural compounds for clinical applications. By integrating these cutting-edge technologies, *Streptomyces*-derived anticancer compounds can be refined for more effective and safer clinical applications. The metagenomics approach also continues to uncover novel biosynthetic gene clusters, providing opportunities for the development of more selective and less toxic anticancer agents.

## Conclusion

Metagenomics has revolutionized the discovery of anticancer compounds from *Streptomyces* spp., enabling the identification of novel biosynthetic pathways and bioactive molecules. This approach accelerates drug discovery by uncovering compounds that were previously inaccessible through traditional culturing methods. However, many of these compounds remain in early research stages, requiring further characterization and validation through rigorous clinical trials. Integrating metagenomics with an AI-driven approach in the discovery of new compounds can accelerate in research and discovery of new compounds from *Streptomyces*. Besides, a novel approach that includes genome editing can also be adopted for anticancer bioprospecting purposes. Additionally, the continued refinement of metagenomic techniques and computational modeling will play a crucial role in overcoming current challenges, such as strain optimization and bioavailability of identified compounds. Collaboration between bioinformaticians, microbiologists, and clinical researchers will be essential to translating laboratory discoveries into viable therapeutic solutions. As metagenomics and synthetic biology continue to evolve, we anticipate an accelerated discovery pipeline that brings novel, more effective anticancer agents to clinical applications, ultimately improving cancer treatment outcomes worldwide. Despite these challenges, metagenomics continues to be a transformative tool, offering a pathway for developing next-generation anticancer therapies and expanding the arsenal of effective treatments against cancer.
